# 709. Risk Factors for *Candida auris* Candidemia: Results from a Multicenter Case-Control Study

**DOI:** 10.1093/ofid/ofab466.906

**Published:** 2021-12-04

**Authors:** Samuel Simon, Rosanna Li, Justin A Andrade, Biju Tharian, Michael Silver, Diana Villanueva Cox, Daniel Gonzalez, Ariel M Mayer, Lung H Fu, James Truong, Nilka Figueroa, Monica Ghitan, Edward Chapnick, Yu Shia Lin

**Affiliations:** 1 Maimondes Medical Center, Brooklyn, New York; 2 Maimonides Medical Center, Brooklyn, New York; 3 The Brooklyn Hospital Center/LIU Pharmacy, Brooklyn, New York; 4 Coney Island Hospital, Brooklyn, New York; 5 The Brooklyn Hospital Center, Brooklyn, New York

## Abstract

**Background:**

The emergence of *Candida auris* as a global pathogen has been described as a serious global threat by the CDC. It has caused outbreaks in healthcare settings as it is transmissible between patients. The risk factors for candidemia caused by *C. auris* may be different than candidemia caused by other *Candida spp*.

**Methods:**

We performed a multicenter, retrospective case-control study at three hospitals in Brooklyn, New York between 2016 and 2020. Patients with at least one positive blood culture for *Candida spp* who were started empirically on an antifungal within 24 hours of blood culture positivity were included in the study. Subsequent cases in the same patient were excluded unless separated by at least 90 days from the initial case. Similar variables such as antibiotics and antifungals within the same drug class were compressed into one variable. Variables with a p-value ≤ 0.05 on univariate analysis were entered into a multivariable analysis with a p-value ≤ 0.05 considered to be statistically significant.

**Results:**

84 cases of *C. auris* candidemia and 105 cases of candidemia caused by other *Candida spp* were included in the analysis. The most common species of other *Candida spp* was *C. glabrata* (N=33, 31.7%) followed by *C. albicans* (N=32, 30.4%). In the multivariable model, the strongest risk factor for *C. auris* candidemia was prior infection or colonization with *C. auris* (aOR 17.5; 95% CI, 1.60-192.93; P = 0.019) followed by prior infection or colonization with multidrug-resistant bacteria (aOR 6.97; 95% CI 1.49-32.74, P = 0.014). A history of peripheral vascular disease (PVD) (aOR 7.78; 95% CI 1.34-45.34, P = 0.023), cerebrovascular disease (CVA) (aOR 4.24; 95% CI 1.18-15.20, P = 0.027) and hemiplegia (aOR 6.43; 95% CI 1.19-34.85, P = 0.031) were also statistically significant. These risk factors remained significant analyzing only patients without any history of *C. auris*.

**Conclusion:**

These data suggest that in hospitalized patients with candidemia, a history of colonization or infection with *C. auris*, prior infection or colonization with multidrug-resistant bacteria, as well as a history of PVD, CVA, and hemiplegia are associated with *C. auris* candidemia.

**Disclosures:**

**Samuel Simon, PharmD**, **Accelerate Diagnostics** (Employee)

